# Visualization of Three-Dimensional SSC (Soluble Solids Content) Across the Entire Surface of Strawberries Using Near-Infrared Hyperspectral Imaging

**DOI:** 10.3390/foods15091563

**Published:** 2026-05-01

**Authors:** Hayato Seki, Bin Li, Tetsuo Kawaide, Te Ma, Satoru Tsuchikawa, Tetsuya Inagaki

**Affiliations:** 1 Institute of Agricultural Machinery, National Agricultural and Food Research Organization, 1-40-2, Nisshin-Cho, Kita-Ku, Saitama 331-8537, Japan; seki.hayato727@naro.go.jp (H.S.);; 2 Graduate School of Bioagricultural Sciences, Nagoya University, Furo-Cho, Chikusa, Nagoya 464-8601, Japan

**Keywords:** strawberry, soluble solids content (SSC), hyperspectral imaging (HSI), near-infrared spectroscopy, geometric correction, 3D shape reconstruction, PLS regression, reliability index, non-destructive quality evaluation

## Abstract

Near-infrared hyperspectral imaging (NIR-HSI) is widely used as a non-destructive technique for evaluating internal fruit quality; however, reliable pixel-wise visualization remains challenging due to geometry-induced spectral distortions and the lack of statistically interpretable validation criteria. This study proposes an integrated framework for three-dimensional visualization of soluble solids content (SSC) across the entire surface of strawberries using NIR-HSI combined with shape-aware spectral correction and pixel-level reliability assessment. Two complementary imaging systems—a line-scan system and a rotation-scan system—were used to acquire hyperspectral and 3D shape data. Fruit height and surface orientation were incorporated into spectral preprocessing to reduce illumination and curvature effects. Partial least squares regression (PLSR) models were developed using region-of-interest-averaged spectra and applied to pixel-wise SSC mapping. To assess the statistical validity of pixel-level predictions, an imaging reliability index based on the Mahalanobis distance in the PLS score space was introduced. The results show that models with high sample-level accuracy do not necessarily produce reliable SSC maps, whereas reliability-based model selection improves image interpretability. This framework enables consistent three-dimensional SSC visualization and is applicable to hyperspectral imaging of internal fruit attributes.

## 1. Introduction

Fruit is one of the most widely consumed foods worldwide, and the fruit industry plays a vital role in many regions. Post-harvest quality inspection and grading support the distribution of fruit with standardized quality, thereby increasing profitability and enhancing market competitiveness. Fruit quality comprises both external and internal attributes. While external traits such as size, color, and shape are important, consumers often place greater emphasis on internal attributes because they directly influence taste [1].

In particular, the strawberry is a kind of very popular fruit with its attractive appearance, unique flavor, and nutritional compositions. Global strawberry production has expanded in acreage over recent decades due to rising demand, driving research efforts to address threats to production while improving fruit quality to meet consumer preferences [2]. Consequently, considerable effort is devoted to developing reliable methods for evaluating strawberry quality.

Non-destructive and high-speed image-based approaches have been widely investigated for evaluating external strawberry quality. Numerous systems have been developed for grading and maturity classification based on geometric and color features extracted from 2D images [3,4,5,6]. However, because these approaches fundamentally rely on surface appearance, they are unable to capture internal quality attribute.

For internal quality assessment, Near-infrared (NIR) spectroscopy has been widely used for non-destructive prediction of soluble solids content (SSC) in strawberries based on point-based spectral measurements [7,8,9]. Hyperspectral imaging (HSI) further extends this approach by enabling pixel-wise analysis and spatial visualization of soluble solids content and other internal quality attributes in strawberries [10]. Strawberries exhibit pronounced spatial heterogeneity in chemical composition: SSC and free sugar levels are substantially higher in the upper fruit region and decrease toward the peduncle, whereas organic acids remain relatively uniform throughout the flesh [11]. Consequently, accurate characterization of internal quality attributes requires region-specific evaluation, which has motivated increasing interest in HSI-based visualization of internal variability. Recent studies have demonstrated that hyperspectral imaging combined with deep learning has been increasingly applied to strawberry quality assessment, including maturity and soluble solids content determination as well as early detection and spatial visualization of quality defects [12,13,14]. HSI has also been applied to the non-destructive evaluation of various strawberry quality attributes, including postharvest quality monitoring under different processing and storage conditions [15].

However, HSI measurements are strongly influenced by fruit geometry—including curvature, variations in optical path length, scattering behavior, and shading—which can substantially degrade prediction accuracy if geometric effects are not accounted for. For small or strongly curved objects, reflectance intensity diminishes toward the edges due to angular effects described by the Lambert cosine law, resulting in pronounced pixel-level inconsistencies. Delwiche et al. [16] identified curvature-induced reflectance variation as a dominant error source in cereal-seed-sized objects and demonstrated that Lambertian correction can partially reduce spatial non-uniformity. Building on this concept, Li et al. [17,18] showed that integrating 3D shape profiles into NIR-HSI improves reflectance uniformity and enhances bruise-detection accuracy in apples by correcting both height- and surface-normal-dependent distortions. Their findings underscore the substantial impact of geometric factors on pixel-wise spectral fidelity. Along this line, the combination of three-dimensional shape measurement and hyperspectral imaging has been proposed as a four-dimensional framework for fruit grading, enabling simultaneous assessment of external geometry and subsurface defects, though the reliability of pixel-wise quantitative imaging has not been explicitly addressed [19].

In parallel, spectral variability within strawberries also arises from region-dependent differences in curvature, scattering properties, and local composition. Chun et al. [20] demonstrated that region-wise segmentation yields markedly different noise characteristics and model performance, with peripheral regions exhibiting larger errors. Moreover, Seki et al. [21] showed that HSI can successfully visualize SSC distribution even in white strawberries, where external coloration provides limited information, further highlighting the potential of NIR-HSI for internal quality mapping. Despite these advances, methodological challenges remain in ensuring the reliability of pixel-wise SSC mapping.

First, neither Li et al. [17] nor Seki et al. [21] quantitatively evaluated whether pixel-wise spectra—either after geometric correction or during visualization—are statistically consistent with the calibration manifold of the predictive model.

Second, conventional performance metrics such as $R^{2}$ and RMSEP are derived from averaged spectra and therefore cannot capture the pixel-level reliability of SSC maps, despite the well-recognized risks of spectral outliers and noise amplification during image generation. Consequently, ensuring the statistical reliability of pixel-wise predictions remains an essential and unresolved challenge in HSI-based internal quality evaluation. Because PLSR models are typically constructed using averaged spectra but applied on a pixel-wise basis, rigorous evaluation of pixel-level reliability is indispensable for robust SSC visualization.

To address these limitations, the purpose of this study is to establish a unified framework for reliable three-dimensional visualization of soluble solids content (SSC) in strawberries using near-infrared hyperspectral imaging.

Unlike previous studies that primarily evaluated model performance using sample-averaged spectra and conventional accuracy metrics, this study focuses on the statistical validity of pixel-wise predictions applied to hyperspectral images. The originality of this work lies in the integration of shape-aware spectral correction with a pixel-level reliability assessment based on the Mahalanobis distance in the partial least squares (PLS) score space, enabling quantitative evaluation of whether imaging spectra are statistically consistent with the calibration manifold. Furthermore, the proposed framework is validated under two different acquisition schemes—a line-scan system and a rotation-scan system—to demonstrate its robustness across measurement geometries. By explicitly addressing both geometry-induced spectral distortion and pixel-wise reliability, this study provides a principled approach for interpretable and reliable hyperspectral visualization of internal fruit quality.

## 2. Materials and Methods

### 2.1. Sample Preparation

In this study, two datasets were prepared using different hyperspectral imaging systems. Dataset I was acquired using a line-scan system, whereas Dataset II was obtained using a rotation-scan system. A total of 193 strawberries for Dataset I and 130 strawberries for Dataset II, all belonging to the “Tochigi i37” cultivar grown in Tochigi Prefecture, Japan, were collected. The samples were transported under refrigerated conditions and stored at 5 °C until one hour before measurement. Before imaging, the strawberries were equilibrated at 23 °C in the laboratory. Some samples exhibited minor damage during transportation, although this did not affect their inclusion in the study. The strawberries used in this study were commercially sourced and measured within two days after transportation. Samples were predominantly at a market-ripe stage with red surface coloration. Because the near-infrared wavelength range employed in this study is not influenced by visible color, surface coloration was not considered a major confounding factor.

### 2.2. Data Acquisition

**Figure 1:**
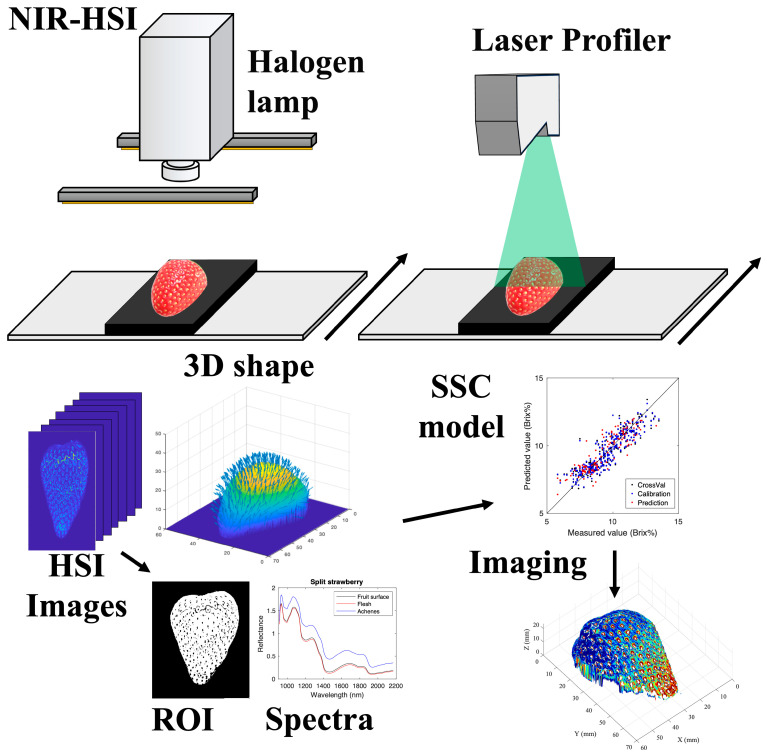
Conceptual workflow of hyperspectral data acquisition and SSC imaging for Dataset I (line-scan system). Near-infrared hyperspectral images and 3D shape data are acquired simultaneously. After background removal and flesh-ROI extraction, geometric correction (height and angle) and spectral preprocessing are applied. PLSR models are constructed using ROI-averaged spectra and subsequently applied to pixel-wise spectra to generate SSC maps, which are finally fused with shape data for three-dimensional visualization. In the SSC imaging results, differences in color correspond to differences in Brix.

**Figure 2:**
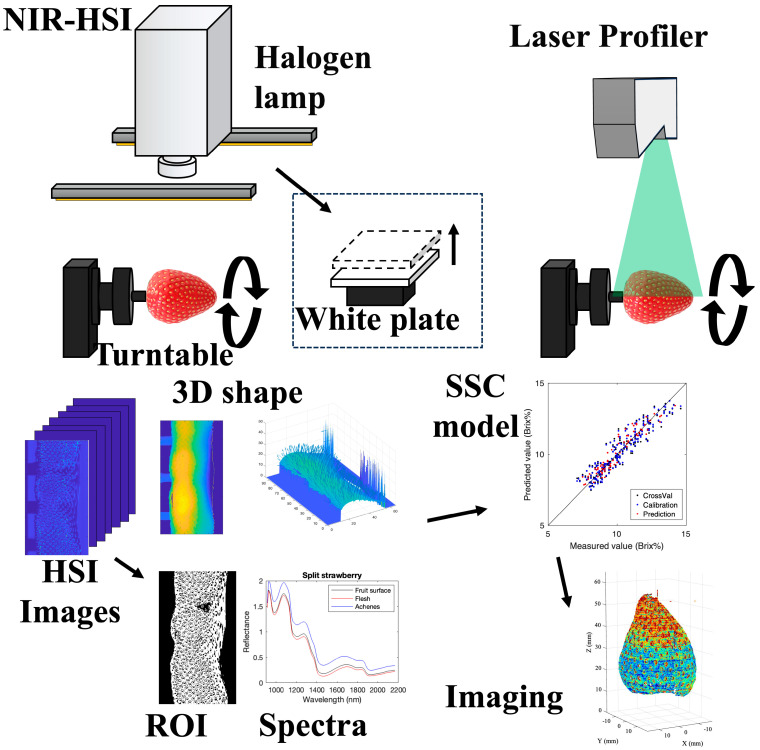
Conceptual workflow of hyperspectral data acquisition and SSC imaging for Dataset II (rotation-scan system). Near-infrared hyperspectral images and 3D shape data are acquired during a full rotation of the fruit on a turntable, enabling full-surface coverage. After background removal and flesh-ROI extraction, geometric correction (height and angle) and spectral preprocessing are applied. PLSR models constructed from ROI-averaged spectra are then applied to pixel-wise spectra to generate SSC maps, which are fused with the reconstructed shape to produce three-dimensional SSC visualization over the entire fruit surface. In the SSC imaging results, differences in color represent differences in Brix.

Figure 1 and Figure 2 illustrate the conceptual workflows for hyperspectral data acquisition and SSC imaging for Datasets I and II, respectively. Dataset I was measured using a line-scan system, whereas Dataset II employed a rotation-scan system to capture hyperspectral images together with corresponding shape information.

Near-infrared hyperspectral images and 3D shape data are acquired during a full rotation of the fruit on a turntable, enabling full-surface coverage. After background removal and flesh-ROI extraction, geometric correction (height and angle) and spectral preprocessing are applied. PLSR models constructed from ROI-averaged spectra are then applied to pixel-wise spectra to generate SSC maps, which are fused with the reconstructed shape to produce three-dimensional SSC visualization over the entire fruit surface.

#### 2.2.1. Systems

The measurement system consisted of a near-infrared hyperspectral imaging (NIR-HSI) system (push-broom line scanning type; Compovision, Sumitomo Electric Industries, Ltd., Tokyo, Japan), a light source, and a laser displacement meter (LJ-X8200, KEYENCE, Ltd., Osaka, Japan). For the line-scan system, sample movement was achieved using a slide table integrated into the NIR-HSI system. In the rotation-scan system, a turntable (OSMS-40YAW, Sigumakoki, Co., Ltd., Tokyo, Japan) was employed. The NIR-HSI system was equipped with a spectroscope and a two-dimensional InGaAs/GaAsSb photosensitive element (256 pixels for wavelength × 320 pixels for position), capable of detecting NIR light in the range of 913–2519 nm at a spectral interval of 6.2 nm. A wavelength ranging from 913 to 2166 nm (i.e., 200 wavelength bands) was selected here because reflectance over 2166 nm has a low signal-to-noise ratio. Illumination was provided by a tube-shaped light source with four halogen lamps positioned on both sides, and the irradiation angle was set to 45°. The laser displacement meter utilized a 405 nm laser, with a profile data count of 3200 points and Z-axis repeatability of 1 μm.

#### 2.2.2. Hyperspectral Images Data and 3D Scan Data Measurement

In the line scan system, the NIR HSI frame rates were set to 150 frames s^−1^ for sample images and 320 frames s^−1^ for both dark and white reference images. Samples were placed on precisely machined aluminum blocks (60 mm × 70 mm) to ensure accurate alignment between the hyperspectral data obtained from the NIR HSI system and the shape data acquired by the laser displacement meter. A black sheet was attached to the block surface to improve background segmentation.

The field of view of both the NIR HSI system and the laser displacement meter was calibrated using a reference rectangular block or landmark. The laser displacement meter operated at a scanning speed of 2.5 mm s^−1^. Dark reference images were acquired with the lens cap closed to prevent light interference. For reference measurements, a white calibration board was placed above the block. The white board was 10mm thick.

In the rotation scan system, the NIR HSI frame rates were set to 100 frames s^−1^ for sample images, 100 and 320 frames s^−1^ for dark images, and 320 frames s^−1^ for white reference images. The NIR HSI system and the laser displacement meter were used to acquire hyperspectral images and shape data during one full rotation of the turntable, which was operated at 30° s^−1^. Reference data were obtained by measuring a white calibration board at height intervals of 2 mm from 0 to 30 mm, using the center of the turntable as the rotational origin. Dark reference images were recorded with the lens cap closed to prevent external light from entering the detector.

#### 2.2.3. SSC Measurement

After hyperspectral imaging, each measurement surface was divided into two regions corresponding to the fruit apex (top) and the fruit base (bottom). Each section was wrapped in a nonwoven cloth, manually squeezed, and pressed to extract juice. The extracted juice was gently stirred, and the soluble solids content (SSC) was measured as Brix using a digital Brix meter (PR-101α, ATAGO Co., Ltd., Tokyo, Japan).

### 2.3. Data Analysis

#### 2.3.1. Geometric Correction and Spectral Preprocessing

Shape correction is an important preprocessing step in hyperspectral imaging of fruits and other agricultural products, as sample geometry strongly influences the measured reflectance spectra. A widely used approach for shape correction is Lambertian surface correction, which assumes that the sample surface behaves as an ideal diffuse reflector. According to the Lambertian model, the reflected intensity decreases in proportion to the cosine of the angle between the surface normal and the observation direction. This model and its variants have been extensively applied to compensate for curvature-related effects and non-uniform illumination in hyperspectral imaging of spherical fruits [16,17].

This study evaluated a preprocessing method for hyperspectral data using 3D profile data and its effectiveness. The data was first cut out based on the measurement block to match the coordinates of the measured hyperspectral data and shape data. In addition, the shape data was resized based on the data size of the hyperspectral data.

The reflectance of each pixel $\left(i,j\right)$ at each wavelength was calculated using a general formula without employing geometric shape correction by Equation (1).

(1) $$R_{\lambda }\left(i,j\right)=\frac{I_{\lambda }\left(i,j\right)-D_{\lambda }\left(i\right)}{W_{\lambda }\left(i\right)-D_{\lambda }\left(i\right)}$$

where λ, i and j represent the wavelength and pixel index variables; $R_{\lambda }\left(i,j\right)$ represents the standardized reflectance intensity at wavelength λ and pixel; $I_{\lambda }\left(i,j\right)$, $W_{\lambda }\left(i\right)$ and $D_{\lambda }\left(i\right)$ represent sample, white reference images and dark images, respectively.

In Dataset I, the height-corrected sample intensity ${I^{\prime }}_{h,\lambda }\left(i,j\right)$ data for each pixel was calculated from Equation (2), employing a simplified geometric attenuation model. This model approximates distance-dependent intensity variation by referencing the inverse square law, without explicitly modeling complex light scattering and illumination geometry. The diffuse light source was therefore treated as providing vertically downward illumination.

(2) $${{I^{\prime }}_{h,\lambda }\left(i,j\right)}^{*}=\frac{I_{\lambda }\left(i,j\right)-D_{\lambda }\left(i\right)}{{\left(\frac{H_{1}+H_{s}\left(i,j\right)}{H_{1}}\right)}^{2}\cdot {\left(\frac{H_{2}+H_{s}\left(i,j\right)}{H_{2}}\right)}^{2}}$$

where $H_{1}$ is the distance from the light source to the white panel surface, $H_{2}$ is the distance from the camera lens to the white panel surface, and $H_{s}$ is the height data of each pixel with the white panel surface height of 0 mm.

The distance $H_{1}$ was 100 mm, and the distance $H_{2}$ was 200 mm. For Dataset II, the brightness values of the white plate measured at discrete heights were used to calculate *${I^{\prime }}_{h,\lambda }\left(i,j\right)$. The height of each pixel was determined from the 3D scan information, and the corresponding white reference values were obtained by linear interpolation along the height direction based on the measured white plate data. Equation (3) was used to calculate the height-corrected relative reflectance $\left({R^{\prime }}_{h,\lambda }\right)$.

(3) $${R^{\prime }}_{h,\lambda }\left(i,j\right)=\frac{{I^{\prime }}_{h,\lambda }\left(i,j\right)}{W_{\lambda }\left(i\right)-D_{\lambda }\left(i\right)}$$

The angle(*θ*) between the surface normal vector and the vertical vector was calculated using the height information of the pixels in the width direction. Equation (4) was applied to obtain the intensity data of the corrected sample $\left({I^{\prime }}_{a,\lambda }\right)$ by applying an angle correction using Lambertian.

(4) $${I^{\prime }}_{a,\lambda }\left(i,j\right)=\frac{I_{\lambda }\left(i,j\right)-D_{\lambda }\left(i\right)}{\cos\theta \left(i,j\right)}$$

The relative reflectance corrected by Lambertian $\left({R^{\prime }}_{a,\lambda }\right)$ was calculated using Equation (5).

(5) $${R^{\prime }}_{a,\lambda }\left(i,j\right)=\frac{{I^{\prime }}_{a,\lambda }\left(i,j\right)}{W_{\lambda }\left(i\right)-D_{\lambda }\left(i\right)}$$

Equation (6) was used to calculate the intensity data for each pixel with height and angle corrections $\left({I^{\prime }}_{h\_a,\lambda }\right)$.

(6) $${I^{\prime }}_{h\_a,\lambda }\left(i,j\right)=\frac{{I^{\prime }}_{h,\lambda }\left(i,j\right)}{\cos\theta \left(i,j\right)}$$

The relative reflectance corrected height and angle corrections $\left({R^{\prime }}_{h\_a,\lambda }\right)$ was calculated using Equation (7).

(7) $${R^{\prime }}_{h\_a,\lambda }\left(i,j\right)=\frac{{I^{\prime }}_{h\_a,\lambda }\left(i,j\right)}{W_{\lambda }\left(i\right)-D_{\lambda }\left(i\right)}$$

These preprocessing steps were applied uniformly to both calibration and imaging data to reduce geometry-induced spectral variability, thereby providing a consistent basis for subsequent ROI averaging, model construction, and pixel-wise reliability evaluation.

#### 2.3.2. Region of Interest (ROI) Determination

To create an SSC estimation model, it is necessary to determine the region of interest (ROI) and calculate the average spectrum. ROI was determined by excluding pixels containing achenes from the fruit surface pixels, using A method combining PCA and image processing [21]. Specifically, we first created an ROI mask by removing the background outside the fruit using thresholding. We then calculated loading vectors for each pixel within the ROI range using principal component analysis. Using these loading vectors, we computed the PC1 score for each pixel. Finally, we created an ROI mask by removing pixels outside the fruit surface from the entire image through binarization using Otsu’s method [22]. For Dataset II, the ROI for the fruit region was determined using a reflectance image at 1204 nm with a threshold of 0.2 for background processing. For Dataset II, the ROI was determined using a reflectance intensity image at 1123 nm with a threshold of 12,000. For PCA, data within the wavelength range 1160–2168 nm was used for Dataset II and data within the wavelength range 1476–1917 nm was used for Dataset II. These parameters were determined through preliminary analysis. The average spectra of the fruit top and fruit bottom for each sample within this defined ROI were calculated.

#### 2.3.3. Spectral Preprocessing

Based on the ROI-averaged spectra obtained as described above, spectral preprocessing was applied prior to PLSR model construction. Spectral processing is generally considered when developing quantitative models to remove noise and disturbances from spectral data and improve prediction accuracy. In this study, spectral processing methods included no processing, the representative Savitzky–Golay smoothing [23], and eight patterns combining first-order differentiation, second-order differentiation (window size: 9, polyorder: 2), and SNV (Standard Normalize variate). These preprocessed spectra were subsequently used for PLSR model development and evaluation, as described in the following section.

#### 2.3.4. SSC Estimation Model

A model for estimating SSC from the average spectrum using PLSR [24] was created by combining geometric spectral correction and spectral preprocessing to generate 32 patterns. The dataset was split 7:3 into training and test sets for model building and evaluation. The split was performed using SSC-stratified sampling to keep the SSC distributions comparable between the training and test sets. The model construction optimized the number of PLS factors by 5-hold-out cross-validation for the training set. The optimal LVs were selected for the maximum root-mean-square error (RMSE) for cross-validation (RMSECV) within the global minimum + 1 standard deviation range. We used 5-fold cross-validation on the training set to select the optimal number of latent variables (LVs) by the “one-standard-error” rule and reported external test performance on the held-out set. The quality of the PLSR model was assessed using the determination coefficient $R^{2}$ and RMSE for calibration ($R^{2}c$ and RMSEC) and prediction ($R^{2}p$ and RMSEP). A good model possesses a low RMSEC, RMSEP, and high determination coefficient ($R^{2}c,\;R^{2}p$) such that calibration and confirmation results do not diverge.

#### 2.3.5. SSC Imaging

PLSR model described above, pixel-wise SSC imaging was performed for the test dataset. Pixels corresponding to the flesh ROI in the hyperspectral images of the test data were used for sugar content visualization by applying the PLSR model constructed to estimate the SSC values. Because the hyperspectral images and the 3D shape data were acquired simultaneously and subsequently resized into a unified spatial grid, pixel-wise correspondence between spectral and geometric information was preserved, allowing direct fusion of SSC maps and shape data to generate three-dimensional visualizations.

#### 2.3.6. SSC Estimation Model Reliability

Generally, hyperspectral imaging performance is evaluated using global metrics such as the coefficient of determination ($R^{2}$), standard error, residual predictive deviation (RPD), and bias, which are derived from calibration or validation datasets. These metrics are effective for assessing the predictive performance of regression models built from averaged spectra. However, in hyperspectral imaging, the estimated SSC at each pixel is calculated by applying a model developed from the mean spectrum of a region of interest (ROI) to pixel-wise spectra, whose statistical properties may differ substantially from those of the calibration spectra. Furthermore, pixel-level spectra are strongly affected by factors such as local illumination conditions, surface geometry, signal-to-noise ratio, and spectral preprocessing. As a result, different preprocessing strategies may lead to substantial differences in spatial noise patterns and apparent heterogeneity in SSC maps, even when global prediction accuracy metrics remain similar. Therefore, conventional regression metrics alone are insufficient to evaluate the reliability of hyperspectral SSC mapping. To address this limitation, we propose a statistical evaluation framework based on the distribution of pixel-wise PLS scores, which enables quantitative assessment of the consistency between imaging spectra and the calibration dataset.

PLSR decomposes the spectral matrix X and the response variable y as Equation (8) [25]:

(8) $$X={TP}^{⊤}+E,\;y=Tq+f$$

where ⊤ represents the latent score matrix. Let $X_{train}\in R^{m\times \lambda }$ denote the preprocessed spectral matrix of the calibration dataset, where m is the number of calibration samples and λ is the number of wavelengths. The PLS score matrix of the calibration dataset is given by Equation (9).

(9) $$T_{train}=X_{train}W^{*}$$

where $W^{*}=W(P^{⊤}W{)}^{-1}$ and W and P are the PLS weight and loading matrices, respectively. For hyperspectral imaging, each pixel spectrum $x_{j}\in R^{p}$ within the ROI is projected onto the same PLS latent space as Equation (10).

(10) $$t_{j}=x_{j}W^{*}$$

where $t_{j}\in R^{A}$ is the PLS score vector of pixel j.

To evaluate whether a pixel-wise spectrum is statistically consistent with the calibration data, the squared Mahalanobis distance of each pixel score vector is computed in the PLS score space. The mean vector μ and covariance matrix Σ of the calibration score matrix $T_{train}$ are defined as Equation (11).

(11) $$\mu =\frac{1}{m}\sum_{i=1}^{m}t_{i},\;\Sigma =\frac{1}{m-1}\sum_{i=1}^{m}\left(t_{i}-\mu \right){\left(t_{i}-\mu \right)}^{⊤}$$

The squared Mahalanobis distance of pixel *j* is then calculated as Equation (12).

(12) $${D_{j}}^{2}={\left(t_{i}-\mu \right)}^{⊤}\Sigma^{-1}\left(t_{i}-\mu \right)$$

Assuming that the PLS scores of the calibration dataset approximately follow a multivariate normal distribution, $D_{j}^{2}$ follows a chi-square distribution with *A* degrees of freedom as Equation (13).

(13) $${D_{j}}^{2}\sim \chi^{2}\left(A\right)$$

To define a statistically interpretable threshold, a kσ criterion is introduced. The corresponding cumulative probability of a standard normal distribution is given by Equation (14).

(14) $$\alpha \left(k\right)=Pr\left(\left|Z\right|<k\right)=erf\left(\frac{k}{\sqrt{2}}\right)$$

where Z∼N(0, 1). In this study, k=3 was adopted, corresponding to α≈0.997.

The Mahalanobis distance threshold is therefore defined as Equation (15).

(15) $$\tau ={\chi^{2}}_{A,\;a}$$

are classified as inliers, while pixels exceeding the threshold are regarded as outliers in the PLS score space. Let N denote the total number of pixels within the ROI. The imaging reliability index is defined as the proportion of inlier pixels (Equation (16)):

(16) $$Reliability=\frac{1}{N}\sum_{j=1}^{N}1\left({D_{j}}^{2}\leq \tau \right)$$

where *Reliability* is the indicator function.

This index represents the fraction of pixel-wise spectra that are statistically consistent with the calibration dataset under the selected preprocessing and model complexity. A higher Reliability value indicates a more reliable SSC map with reduced influence of spectral distortion and noise amplification. Unlike conventional regression metrics that evaluate prediction accuracy at the sample level, the proposed reliability index directly assesses the statistical validity of pixel-wise predictions in hyperspectral imaging. The pixel-wise reliability index was defined using a *χ*^2^-based boundary in the PLS score space. A 99% $\chi^{2}$ threshold was adopted to define the calibration manifold, providing a conservative yet not overly restrictive criterion for pixel-wise evaluation. This choice avoids arbitrary tuning while preventing excessive rejection of pixel spectra when comparing pixel-wise spectra with the calibration mean spectrum. For intuitive reference, this threshold is approximately comparable to 3σ range in a univariate normal distribution, although the formulation itself is strictly multivariate. The model’s reliability metric was defined as the average value across the test set samples.

All data analyses were conducted using MATLAB 2025a, a computer analysis software package. PLSR were conducted using libPLSR_1.98 [24].

## 3. Results and Discussions

### 3.1. Spectral Analysis

**Figure 3:**
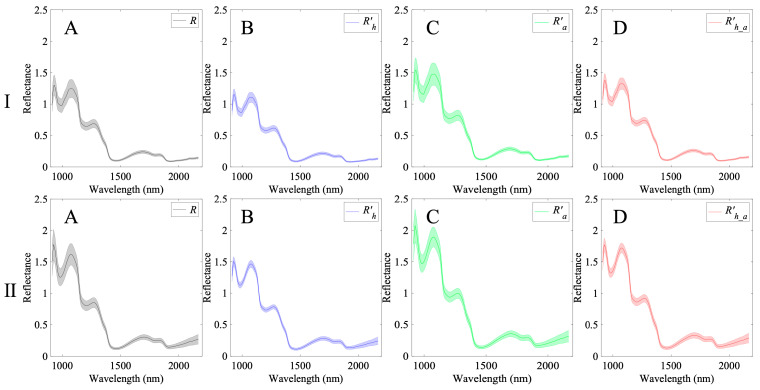
Average reflectance spectra (mean ± standard deviation) of strawberries for Dataset I (line-scan system) and Dataset II (rotation-scan system), shown under four geometric-correction conditions: uncorrected (**A**), height-corrected (**B**), angle-corrected (**C**), and combined height–angle correction (**D**). The shaded areas represent the standard deviation.

Figure 3 shows the average reflectance spectra (*R*) and the average spectra corrected for geometric shape including height $\left({R^{\prime }}_{h}\right)$, angle $\left({R^{\prime }}_{a}\right)$, height and angle $\left({R^{\prime }}_{h\_a}\right)$ for each dataset. The average reflectance spectra had absorption peaks at 970, 1165, 1420, 1780, and 1900 nm. The peaks at 970, 1420, and 1900 nm corresponded to O–H-related water content, those at 1165 and 1780 nm corresponded to C–H-related sugar [26,27]. In the height-corrected spectrum, the standard deviation tended to decrease, particularly in the wavelength band below 1500 nm, suggesting that the effect of distance was reduced for each sample. Regarding angle correction, reflectance tended to be higher overall. This was considered to result from the correction of the angled edges of the fruit. For both height and angle corrections, the characteristics of the effects of both height correction and angle correction were confirmed. Although the height-correction implementations differ between Dataset I and Dataset II, the similar spectral trends observed after correction indicate that both approaches compensate for height-dependent intensity variation. These results suggest that a comparable correction effect can be achieved using Equation (2) without measuring the white reference at multiple heights. Nevertheless, further systematic validation using additional datasets will be required to fully assess reproducibility and model generalizability, which is left for future work.

### 3.2. SSC Models

Table 1 summarizes the dataset. The dataset was split into training and test sets at a 7:3 ratio. To avoid imbalance in SSC distribution between the two sets, samples were stratified based on their reference SSC values and assigned so that the mean and standard deviation of SSC were comparable between the training and test sets. The training set exhibited a wider SSC range and more distributed values. Fruit tops tended to have higher SSC values and larger standard deviations than fruit bottoms, consistent with the general observation that the top of strawberries is sweeter. This trend was consistent across both Dataset I and Dataset II. These dataset characteristics provide the basis for the SSC model evaluation presented below.

Table 2 summarizes the results of the PLSR model performance of the Dataset I. Comparing the coefficients of determination for the test set, the model with smoothing after height correction showed the best prediction accuracy with a coefficient of determination of 0.813 and RMSEP of 0.687 for the test set. This model demonstrates higher estimation accuracy than the uncorrected model, suggesting that geometric shape correction is an effective preprocessing method for the data. Table 3 summarizes the results of the PLSR model performance of Dataset II. The models for all conditions (shape correction and spectral pretreatment) had good prediction accuracy with $R^{2}p$ greater than 0.850. Comparing the coefficients of determination for the test set, the model with SNV-2nd derivative after height correction showed the best prediction accuracy with a coefficient of determination of 0.919 and PMSEP of 0.436 for the test set. Liu et al. [28] demonstrated that suppressing the contribution of spectral components strongly affected by distance can improve prediction accuracy. In the present study, models incorporating distance and angle corrections were likewise selected, supporting the notion that geometric factors play an important role in SSC estimation using NIR-HSI, while the reliability analysis indicates that such geometric considerations do not necessarily guarantee stable pixel-wise predictions under complex acquisition conditions.

### 3.3. SSC Imaging and Reliability

**Figure 4:**
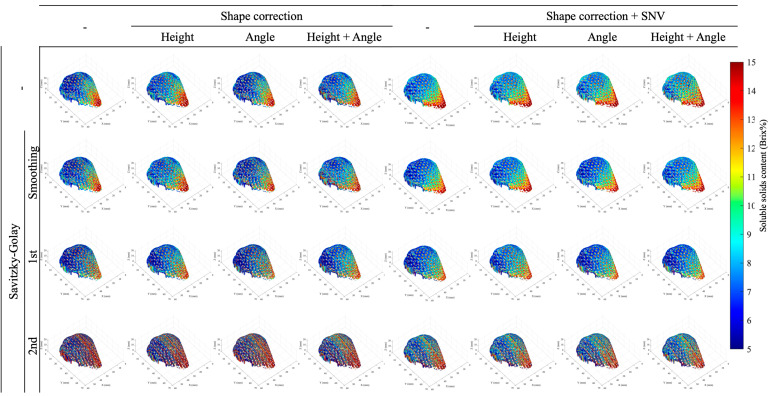
Examples of SSC imaging results for Dataset I acquired using the line-scan system, illustrating the effects of different shape-correction and spectral-processing methods. Each panel displays pixel-wise SSC predictions generated by applying PLSR models under varying preprocessing conditions, highlighting differences in spatial noise, contrast, and image stability.

**Figure 5:**
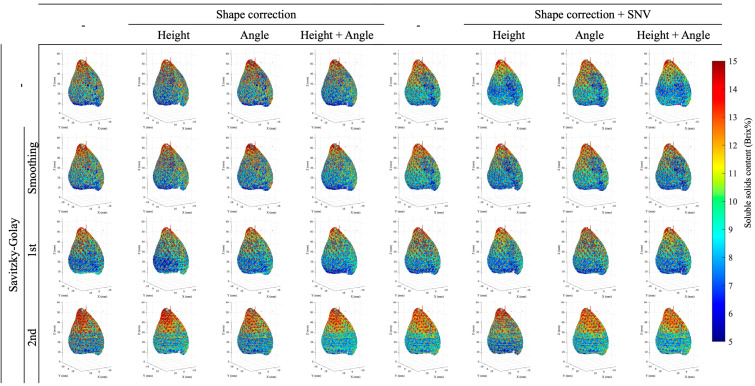
Examples of SSC imaging results for Dataset II acquired using the rotation-scan system, illustrating the effects of different shape-correction and spectral-processing methods. Each panel shows pixel-wise SSC predictions produced by PLSR models under varying preprocessing conditions, highlighting differences in spatial uniformity, noise behavior, and the overall stability of full-surface SSC visualization.

Figure 4 shows the imaging results for geometric correction and spectral processing in Dataset I, while Figure 5 shows the results for Dataset II. The HSI method often employs smoothing filters to reduce noise and improve visibility, but in this case, no filter was used. Both Dataset I and Dataset II demonstrate that the combination of geometric shape correction and spectral processing alters the appearance of the imaging results. In particular, for Dataset I, it can be confirmed that using second-order differentiation processing results in noisy imaging. These visual differences motivate a quantitative evaluation of pixel-wise consistency using the proposed reliability index.

When comparing models using Reliability, the proposed evaluation metric for imaging in this study, the model with the highest coefficient of determination did not necessarily exhibit the highest reliability. In Dataset I, the model using first-derivative spectral processing for height correction achieved 0.554, while in Dataset II, the model using SNV processing and second-derivative processing for both height and angle correction achieved a high value of 0.417. Meanwhile, the reliability of the model with the highest coefficient of determination in Dataset I was 0.012, while the reliability of the model with the highest coefficient of determination in Dataset II was 0.161. This indicates that even with a high model coefficient of determination, many individual pixel spectra predicted from the dataset’s average spectrum are statistically outliers. This reliability metric is likely to be higher with fewer latent variables. However, in Dataset I, with the same number of latent variables, the reliability of the first-order derivative model after height correction was 0.554, while the reliability of the second-order derivative model was 0.290, showing a difference. Second-derivative preprocessing enhanced linear fit at the sample level but also amplified high-frequency noise at the pixel level, widening the score-space dispersion relative to the calibration manifold; this explains the low reliability despite a high test-set $R^{2}$. SNV tends to normalize individual spectra and reduce pixel-wise dispersion relative to the calibration data, which can be advantageous in some datasets. The reliability index is intended to provide a relative measure for comparing pixel-wise consistency among different preprocessing strategies, rather than to define an absolute threshold of image validity. Although Dataset II exhibited higher model-level accuracy, the lower pixel-wise reliability suggests that acquisition geometry, particularly scan direction and fruit rotation, may increase pixel-level spectral dispersion and affect imaging stability.

In hyperspectral imaging, calibration models are typically constructed using averaged spectra, whereas imaging involves pixel-wise spectra acquired under heterogeneous noise and illumination conditions. Consequently, spectral variability arising from complex fruit geometry and measurement conditions may be suppressed during averaging at the model-construction stage, while becoming critical for image-level interpretation. In agricultural products with complex shapes, it is therefore common to examine a wide range of preprocessing strategies. In this study, representative and commonly used preprocessing methods were systematically evaluated. The results indicate that combining geometric correction with appropriate spectral preprocessing improves the proposed reliability index in both datasets, suggesting that such preprocessing can mitigate spectra that deviate substantially from the calibration manifold. These findings imply that pixel-wise application of geometric correction and spectral preprocessing is effective for preserving image validity, as sample-derived spectral variability associated with complex geometry may otherwise be lost during spectral averaging.

**Figure 6:**
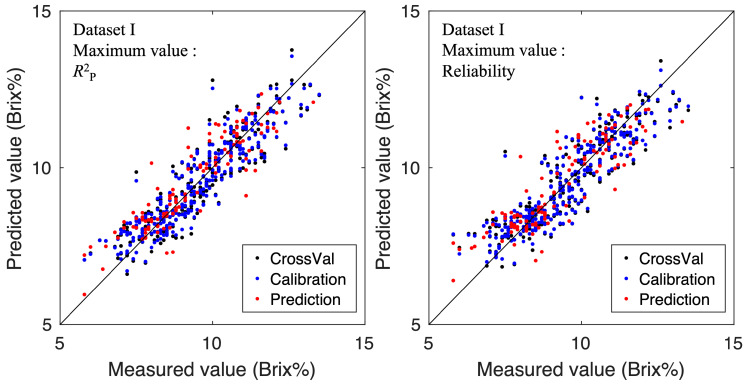
Relationships between predicted SSC values and reference SSC measurements for Dataset I. The left panel shows the model with the highest coefficient of determination ($R^{2}$) on the test set; the right panel shows the model with the highest pixel-wise reliability index.

**Figure 7:**
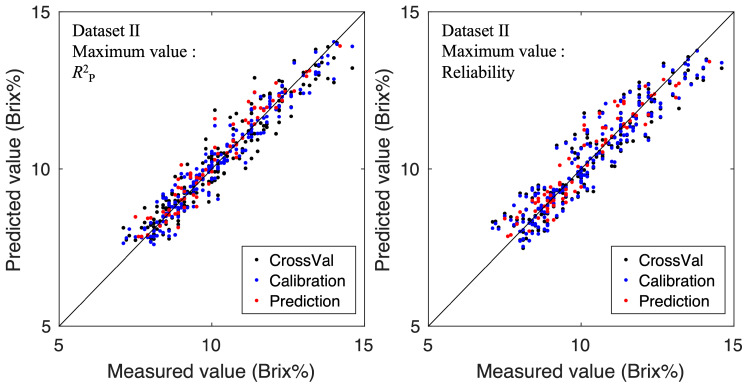
Relationships between predicted SSC values and reference SSC measurements for Dataset II. The left panel illustrates the model with the highest test-set $R^{2}$, while the right panel displays the model with the highest imaging-reliability index.

Figure 6 and Figure 7 show the relationship between the predicted values and actual measured values for the SSC models of Dataset I and Dataset II, respectively. The left panel shows the model with the maximum coefficient of determination in the test set, while the right panel shows the model with the maximum imaging reliability index (Reliability). Both Dataset I and Dataset II showed that the model with the highest coefficient of determination provided the best linear fit. However, even the model with the highest reliability fitted the linear trend sufficiently well, suggesting it could predict values from low to high sugar content. The achieved prediction performance is comparable to that reported in previous HSI-based SSC studies on strawberries [10,20,21]. Therefore, we decided to adopt the model with the highest reliability metric as the imaging model.

**Figure 8:**
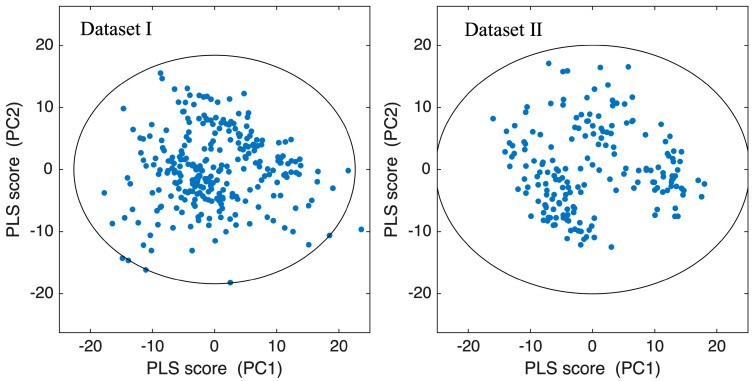
PLS score distributions (LV1 and LV2) of the training datasets for the models with the highest reliability in Dataset I and Dataset II. The ellipse represents the χ^2^-based 99% boundary estimated from the calibration scores, defining the calibration manifold used for pixel-wise reliability evaluation. This figure is provided as a sanity check to confirm that the PLS score distributions do not exhibit strong directional bias or anomalous clustering.

Figure 8 shows a scatter plot of the PLS scores (PC1 and PC2) for the training dataset of the model with the highest reliability for both Dataset I and Dataset II. The calibration scores formed compact distributions without strong directional bias or anomalous clustering, supporting the practical use of a distance-based criterion in the latent score space. To verify the robustness of the proposed reliability-based model selection, we confirmed that the selected model remained unchanged even when the threshold (corresponding to levels of approximately 2σ, 3σ, and 4σ) was altered. These results indicate that the proposed framework is not sensitive to reasonable variations in the threshold and that model selection is driven by intrinsic consistency within the score space rather than arbitrary parameter choices.

### 3.4. 3D SSC Imaging Results

**Figure 9:**
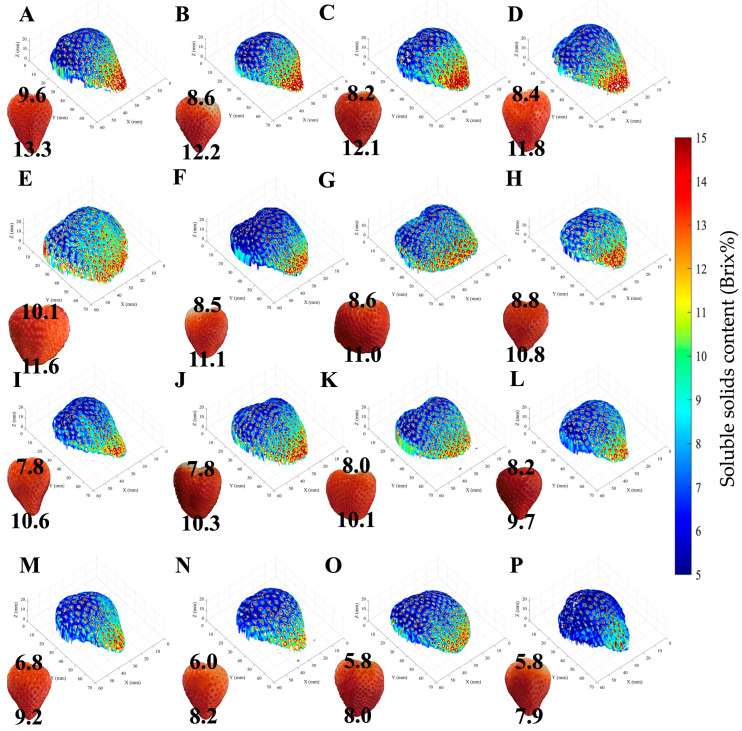
3D SSC imaging results for strawberry samples in the Dataset I test set. Samples are arranged from high to low measured SSC (**A**–**P**). The values in the image indicate the measured Brix values for top and bottom.

**Figure 10:**
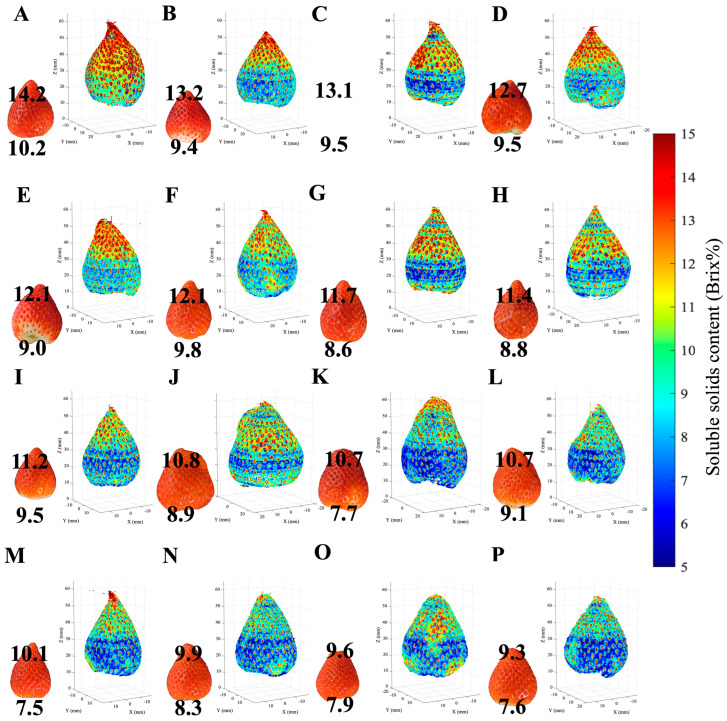
3D SSC imaging results for Dataset II (rotation-scan) test samples. Samples are arranged from high to low measured SSC (**A**–**P**). Note: Panel (**C**) was excluded due to imaging artifacts during acquisition. The values in the image indicate the measured Brix values for top and bottom.

Figure 9 shows the imaging results for the strawberry samples in the test set of Dataset I, and Figure 10 shows the imaging results for the strawberry samples in the test set of Dataset II. The sample alphabet is arranged in descending order of measured SSC, from A to P.

In the imaging data for Dataset I, the SSC values at the fruit top were high for most samples, with those of A, B, C, and D being particularly high and clearly visible. The fruit bottom showed less variation in SSC values compared to the fruit top and tended to be lower. Sample P, in particular, exhibited low sugar content overall; while appearing red in the image, it exhibited consistently low SSC values across the fruit. These findings align with actual measurement results indicating higher sugar content in the strawberry fruit top, lower sugar content in the fruit bottom, and smaller standard deviations. Furthermore, by synthesizing shape data with sugar content imaging, shape can also be evaluated simultaneously. This imaging technique can be applied as a line sorting system for strawberries placed on a container without overlapping fruits. In the imaging data of Dataset II, differences in SSC values were observed between the top and bottom of the fruit, as seen in A (high overall SSC values) and P (low overall SSC values). It should be noted that the reference SSC measurements in this study were limited to the top and bottom regions of the fruit, which restricts direct validation of pixel-wise spatial accuracy across the entire surface. While the high correlations between these reference values and the corresponding ROI-averaged imaging results (Table 2 and Table 3) support the consistency of the SSC maps at the sample-average level, they do not provide direct evidence for fine-scale spatial accuracy within the maps. Validation experiments using samples with known internal heterogeneity would enable a more rigorous assessment of spatial fidelity. Such investigations are considered important for further strengthening the reliability and generalizability of the proposed 3D SSC visualization framework.

By first excluding achene regions and focusing on the flesh ROI, clearer SSC distribution patterns can be obtained, as shown in our previous work [21]. Building on this improvement in two-dimensional visualization, the present study further integrates shape information to enable three-dimensional SSC mapping over the entire fruit surface. This technology, which can measure the sugar content of the entire fruit rather than just one side, holds promise for use in new variety development and as a quality evaluation technique for sorting.

While the present study focuses on soluble solids content as a representative internal quality attribute, comprehensive quality indices integrating multiple physicochemical and sensory-related parameters have been proposed to better represent overall strawberry quality and can be predicted using hyperspectral imaging [29].

## 4. Conclusions

To address the challenge of ensuring reliable pixel-wise predictions in hyperspectral fruit quality evaluation, this study demonstrates that combining geometric correction with pixel-wise reliability assessment improves the interpretability of hyperspectral SSC imaging. Unlike conventional approaches that rely solely on sample-level accuracy metrics, the proposed framework explicitly evaluates the statistical consistency of imaging spectra. By incorporating fruit shape information—specifically height and tilt—into spectral preprocessing, the proposed approach enables reliable three-dimensional visualization of SSC distributions. The introduction of a pixel-level reliability index based on the Mahalanobis distance in the PLS score space provides a robust measure for assessing spatial noise and outliers, with practical implications for fruit quality evaluation, breeding, and sorting systems.

In summary, the proposed framework provides a practical foundation for reliable 3D SSC visualization and offers a promising direction for advanced non-destructive fruit quality assessment. While the present study demonstrates the feasibility and interpretability of the proposed approach, several limitations should be acknowledged. Although pixel-wise SSC prediction is discussed, the reference SSC values were obtained from region-averaged measurements of halved fruits, which limits the ability to claim pixel-level spatial accuracy of the estimated SSC distributions. Additional limitations include the use of instrument-specific acquisition configurations. Future work will consider the extension of this framework toward practical imaging systems, improved spatial validation strategies, and more comprehensive quality evaluation by integrating multiple internal attributes.

## Figures and Tables

**Table 1:** Overview of the datasets used for PLSR model development. Dataset I was measured using a line scan system, while Dataset II was measured using a rotational scan system. The n value indicates the total number of strawberry samples in the dataset and the number of samples in the split training and test sets. SSC represents the distribution of the range and mean ± standard deviation at the top and bottom of the fruit.

Dataset		n	Fruit Section	Range (Brix%)	Mean ± SD (Brix%)
I	Total	193	Bottom	5.8–10.1	8.3 ± 0.91
			Top	7.5–13.5	10.7 ± 1.08
	Train	136	Bottom	5.8–10.1	8.3 ± 0.91
			Top	7.5–13.5	10.8 ± 1.08
	Test	57	Bottom	5.8–10.1	8.1 ± 0.91
			Top	7.9–13.3	10.6 ± 1.08
II	Total	130	Bottom	7.1–11.0	8.9 ± 0.78
			Top	9.2–14.6	11.5 ± 1.19
	Train	93	Bottom	7.1–11.0	8.9 ± 0.82
			Top	9.2–14.6	11.6 ± 1.23
	Test	37	Bottom	7.7–10.2	8.9 ± 0.66
			Top	9.3–14.2	11.4 ± 1.08

**Table 2:** Summary of PLSR performance and pixel-wise imaging reliability for Dataset I acquired using the line-scan system, comparing models under different shape-correction and spectral-processing conditions. The highlights indicate the conditions with the maximum coefficient of determination and the maximum reliability index, respectively.

Spectra	Spectral Processing	LV	RMSECV	RMSEC	RMSEP	*R* ^2^_CV_	*R* ^2^_C_	*R* ^2^_P_	Reliability	r*
*R*	-	6	0.710	0.691	0.738	0.795	0.807	0.785	0.029	0.891
*R*	Smoothing	6	0.680	0.661	0.736	0.812	0.823	0.786	0.031	0.893
*R*	1st derivative	4	0.682	0.658	0.793	0.812	0.824	0.751	0.309	0.872
*R*	2nd derivative	4	0.644	0.614	0.699	0.832	0.847	0.806	0.104	0.916
*R*	SNV	6	0.674	0.645	0.778	0.816	0.831	0.761	0.217	0.887
*R*	SNV-Smoothing	5	0.705	0.676	0.820	0.799	0.814	0.734	0.227	0.867
*R*	SNV-1st derivative	3	0.705	0.684	0.827	0.799	0.81	0.729	0.448	0.849
*R*	SNV-2nd derivative	3	0.654	0.626	0.734	0.826	0.841	0.787	0.211	0.872
*R*′_h_	-	7	0.694	0.652	0.741	0.805	0.828	0.783	0.027	0.896
*R*′_h_	Smoothing	7	0.667	0.631	0.727	0.82	0.839	0.791	0.024	0.900
*R*′_h_	1st derivative	3	0.723	0.700	0.792	0.788	0.801	0.751	0.554	0.874
*R*′_h_	2nd derivative	3	0.754	0.742	0.755	0.77	0.777	0.775	0.290	0.894
*R*′_h_	SNV	6	0.649	0.623	0.739	0.829	0.843	0.784	0.196	0.900
*R*′_h_	SNV-Smoothing	6	0.689	0.658	0.791	0.808	0.824	0.752	0.202	0.883
*R*′_h_	SNV-1st derivative	4	0.706	0.627	0.726	0.798	0.841	0.791	0.323	0.896
*R*′_h_	SNV-2nd derivative	3	0.666	0.636	0.732	0.82	0.836	0.788	0.222	0.902
*R*′_a_	-	6	0.705	0.683	0.715	0.798	0.811	0.798	0.025	0.899
*R*′_a_	Smoothing	6	0.679	0.657	0.707	0.813	0.825	0.802	0.028	0.901
*R*′_a_	1st derivative	4	0.674	0.649	0.764	0.816	0.829	0.769	0.311	0.882
*R*′_a_	2nd derivative	3	0.784	0.763	0.77	0.751	0.764	0.765	0.252	0.890
*R*′_a_	SNV	6	0.653	0.626	0.751	0.827	0.841	0.776	0.202	0.894
*R*′_a_	SNV-Smoothing	5	0.714	0.685	0.823	0.793	0.810	0.732	0.229	0.870
*R*′_a_	SNV-1st derivative	3	0.719	0.694	0.836	0.791	0.804	0.723	0.470	0.862
*R*′_a_	SNV-2nd derivative	2	0.758	0.744	0.773	0.767	0.775	0.764	0.463	0.886
*R*′_h_a_	-	7	0.670	0.635	0.720	0.818	0.837	0.795	0.014	0.904
*R*′_h_a_	Smoothing	7	0.630	0.600	0.687	0.839	0.854	0.813	0.012	0.911
*R*′_h_a_	1st derivative	3	0.719	0.696	0.768	0.79	0.803	0.767	0.448	0.883
*R*′_h_a_	2nd derivative	3	0.738	0.679	0.71	0.779	0.813	0.801	0.209	0.909
*R*′_h_a_	SNV	6	0.632	0.598	0.711	0.838	0.855	0.8	0.178	0.907
*R*′_h_a_	SNV-Smoothing	6	0.682	0.655	0.787	0.811	0.826	0.755	0.21	0.886
*R*′_h_a_	SNV-1st derivative	3	0.728	0.703	0.847	0.785	0.8	0.716	0.501	0.861
*R*′_h_a_	SNV-2nd derivative	3	0.670	0.639	0.738	0.818	0.834	0.784	0.235	0.903

r*: The correlation coefficient represents the linear relationship between the reference SSC values and the imaging-based SSC values calculated as the average of pixel-wise predictions in the top and bottom regions.

**Table 3:** Summary of PLSR performance and pixel-wise imaging reliability for Dataset II acquired using the rotation-scan system, comparing models under different shape-correction and spectral-processing conditions. The highlights indicate the conditions with the maximum coefficient of determination and the maximum reliability index, respectively.

Spectra	Spectral Processing	LV	RMSECV	RMSEC	RMSEP	*R* ^2^_CV_	*R* ^2^_C_	*R* ^2^_P_	Reliability	r*
*R*	-	7	0.508	0.460	0.509	0.907	0.924	0.889	0.026	0.941
*R*	Smoothing	7	0.487	0.445	0.487	0.915	0.929	0.899	0.028	0.946
*R*	1st derivative	6	0.499	0.462	0.520	0.910	0.923	0.885	0.227	0.949
*R*	2nd derivative	5	0.531	0.475	0.464	0.899	0.919	0.908	0.220	0.955
*R*	SNV	8	0.471	0.430	0.485	0.920	0.933	0.900	0.058	0.960
*R*	SNV-Smoothing	8	0.482	0.440	0.498	0.917	0.930	0.894	0.059	0.959
*R*	SNV-1st derivative	7	0.447	0.411	0.475	0.928	0.939	0.904	0.162	0.961
*R*	SNV-2nd derivative	4	0.531	0.547	0.458	0.899	0.892	0.911	0.354	0.957
*R*′_h_	-	8	0.498	0.456	0.522	0.911	0.925	0.884	0.030	0.945
*R*′_h_	Smoothing	8	0.486	0.449	0.503	0.915	0.927	0.892	0.027	0.949
*R*′_h_	1st derivative	7	0.490	0.451	0.474	0.914	0.927	0.904	0.152	0.955
*R*′_h_	2nd derivative	4	0.602	0.545	0.474	0.870	0.893	0.904	0.267	0.943
*R*′_h_	SNV	7	0.544	0.540	0.589	0.893	0.895	0.852	0.145	0.933
*R*′_h_	SNV-Smoothing	8	0.487	0.446	0.544	0.915	0.929	0.874	0.080	0.949
*R*′_h_	SNV-1st derivative	7	0.458	0.426	0.490	0.925	0.935	0.898	0.173	0.950
*R*′_h_	SNV-2nd derivative	4	0.506	0.403	0.436	0.908	0.942	0.919	0.161	0.958
*R*′_a_	-	7	0.542	0.495	0.488	0.894	0.912	0.898	0.043	0.946
*R*′_a_	Smoothing	7	0.515	0.472	0.479	0.905	0.920	0.902	0.042	0.949
*R*′_a_	1st derivative	7	0.511	0.462	0.500	0.906	0.923	0.893	0.138	0.953
*R*′_a_	2nd derivative	5	0.571	0.529	0.488	0.883	0.899	0.898	0.240	0.952
*R*′_a_	SNV	8	0.480	0.438	0.497	0.917	0.931	0.895	0.059	0.958
*R*′_a_	SNV-Smoothing	8	0.490	0.447	0.506	0.914	0.928	0.891	0.059	0.958
*R*′_a_	SNV-1st derivative	7	0.452	0.416	0.482	0.927	0.938	0.901	0.160	0.961
*R*′_a_	SNV-2nd derivative	4	0.557	0.550	0.455	0.888	0.891	0.912	0.363	0.959
*R*′_h_a_	-	8	0.512	0.449	0.516	0.906	0.928	0.886	0.069	0.940
*R*′_h_a_	Smoothing	7	0.546	0.448	0.497	0.893	0.928	0.895	0.064	0.944
*R*′_h_a_	1st derivative	7	0.520	0.492	0.497	0.903	0.913	0.895	0.188	0.944
*R*′_h_a_	2nd derivative	4	0.632	0.595	0.528	0.856	0.872	0.881	0.330	0.938
*R*′_h_a_	SNV	7	0.585	0.567	0.577	0.877	0.884	0.858	0.149	0.932
*R*′_h_a_	SNV-Smoothing	8	0.495	0.451	0.556	0.912	0.927	0.868	0.080	0.942
*R*′_h_a_	SNV-1st derivative	7	0.466	0.433	0.494	0.922	0.932	0.896	0.175	0.944
*R*′_h_a_	SNV-2nd derivative	3	0.611	0.575	0.497	0.866	0.881	0.895	0.417	0.947

r*: The correlation coefficient represents the linear relationship between the reference SSC values and the imaging-based SSC values calculated as the average of pixel-wise predictions in the top and bottom regions.

## Data Availability

The datasets generated and analyzed during the current study are not publicly available due to restrictions related to instrument-specific configurations but are available from the corresponding author on reasonable request.
